# Editorial to Special Issue—Anti-Inflammatory Activity of Natural Products

**DOI:** 10.3390/molecules25081926

**Published:** 2020-04-21

**Authors:** Maria Graça Miguel

**Affiliations:** Faculdade de Ciências e Tecnologia, Mediterranean Institute for Agriculture, Environment and Development, Universidade do Algarve, Campus de Gambelas, 8005-139 Faro, Portugal; mgmiguel@ualg.pt

Inflammation involves several steps to respond to harmful stimuli. Plants, plant products, marine compounds, mushrooms and bee products (honey, propolis, bee pollen) have been a target of study in order to find effective anti-inflammatory compounds of natural origin. This Special Issue aims at unravelling some mechanisms involved in the anti-inflammatory activity of some phenols, terpenes and alkaloids among other secondary metabolites, isolated predominantly from plants and mushrooms.

Kong et al. studied the anti-inflammatory activity of 5-HMF (5-hydroxymethylfurfural) in lipopolysaccharide-stimulated RAW 264.7 cells as well as the mechanisms involved in this action. 5-HMF that occurs in various food products, such as coffee, dried fruits, honey and fruit juices and results from an acid-catalyzed thermal dehydration of fructose in a heterocyclic Maillard reaction, was able to inhibit the production of nitric oxide (NO), prostagandin E2 (PGE2), tumor necrosis factor - α (TNF-α), interleukin-6 (IL-6), IL-1, reactive oxygen species (ROS) by suppressing the protein phosphorylation of MAPK (mitogen activated protein kinases), NF-κB (nuclear factor-κB) and Akt/mTOR (Serine/Threonine kinase / mamalian target of rapamycin) signal pathways in lipopolysaccahride (LPS)-stimulated RAW264.7 macrophage cells [1].

Szebeni et al. studied the anti-inflammatory action of synthetic curcumin analogs, in order to improve the poor bioavailability of curcumin extracted from turmeric that also shows anti-inflammatory activity but without clinical application. The results showed that the curcumin analogs N-((E)-5-(3,5-dihydroxyphenyl)-2-((E)-3-(3,5-dihydroxyphenyl)acryloyl)-3-oxo-1-phenylpent-4-en-1-yl)acetamide and N-((E)-5-(3-hydroxyphenyl)-2-((E)-3-(3-hydroxyphenyl) acryloyl)-3-oxo-1-phenylpent-4-en-1-yl)acrylamide in rat models of TNBS [(2,4,6-trinitrobenzenesulphonic acid)]-induced colitis were able to reduce tissue destruction as well as the inflammatory conditions of submucosal layers, the severity of colonic inflammation and colonic myeloperoxidase (MPO) enzyme activity. The authors also reported that the induction of NF-κB by LPS, *Escherichia coli* O111:B4 in an NF-κB -driven luciferase expressing reporter cell line, was inhibited by those curcumin analogs on a concentration-dependent manner. N-((E)-5-(3-hydroxyphenyl)-2-((E)-3-(3-hydroxyphenyl) acryloyl)-3-oxo-1-phenylpent-4-en-1-yl)acrylamide also inhibited the expression of TNF-α, (IL-6), IL-4 in peripheral blood mononuclear cells (PBMCs) after LPS stimulation [2].

Wang et al. revealed that erinacine C, a cyathane type diterpenoid, extracted from *Hericium erinaceus* fungus that is consumed by Chinese and Japanese people, is able to induce the synthesis of nerve growth factor and has anti-inflammatory activity, particularly anti-neuroinflammatory activity. According to the authors [3], this property is due to the capacity for reducing the levels of NO, IL-6, TNF-α and inducible nitric oxide synthase (iNOS), for inhibiting the expression of NF-κB and phosphorylation of IκBα (p-IκBα) produced by LPS- induced human BV2 microglial cells and for enhancing the nuclear transcription factor erythroid 2-related factor (Nrf2) and the expression of the heme oxygenase-1 (HO-1) [3].

Ye et al. reported that chlojaponilactone B, a lindenane-type sesquiterpenoid extracted from *Chloranthus japonicus* has anti-inflammatory activity. This species has been used in traditional Chinese medicine for treating fractures, neurasthenia, pulmonary tuberculosis, rheumatic arthralgia and traumatic injuries. The authors have shown that the mechanisms involved in the anti-inflammatory activity of that sesquiterpenoid included the reduction of the expression of iNOS, NO, Il-6, TNF-α and COX-2 (cyclooxigenase - 2), the inhibition of phosphorylation of p65 and IκBα and decrease of ROS generation in LPS-induced RAW264.7 murine macrophages. Chlojaponilactone B was able to inhibit the expression 16 April 2020of NF-κB and pro-inflammatory mediators in LPS-induced RAW264.7 murine macrophage cell line by inhibition of toll like receptor 4 (TLR4) and myeloid differentiation factor 88 (MyD88) activation. Molecular docking studies showed that this compound bound to TLR4 in a similar manner to that of TAK-242, a TLR4 inhibitor [4].

Two saponins, polyphyllin VII [5] and astragaloside IV [6], extracted from rhizome of *Paris polyphylla* var. *yunnanensis* and *Astragalus membranaceus* (Fisch.) Bunge, respectively, were reported as having anti-inflammatory activity. Zhang et al. [5] showed that polyphyllin VII, a steroidal saponin in lipopolysaccharide (LPS)-stimulated RAW264.7 cells was able to reduce the production of NO and PGE2 and the protein and mRNA expressions of pro-inflammatory cytokines (TNF-α, IL-1β and IL-6) and enzymes (iNOS, COX-2 and matrix metalloproteinase-9 [MMP-9]) by suppressing the NF-κB and MAPKs pathways. The same saponin also had in vivo capacity for inhibiting xylene-induced ear edema and cotton pellet-induced granuloma formation in mice and suppressing LPS and CuSO4-induced inflammation and toxicity in zebrafish embryos. Wang et al. studied the in vivo anti-inflammatory effect of astragaloside IV as well as the mechanisms involved. For this study, the authors [6] used female mice that were previously treated with astragaloside IV followed by the LPS and an intrauterine perfusion of sodium chloride. From this experiment, the authors [6] observed a reduction of the levels of IL-1β, TNF-α, NO and MPO activity in mice uteri and a suppression of NF-κB, p38 and JNK (c-Jun N-terminal kinase) signal pathways. The authors conclude that astragaloside IV can attenuate LPS-induced endometritis by inhibiting those inflammatory cytokines.

Flavonoids are polyphenols largely spread in plants, which are ingredients in fruits, herbal medicines and teas [7]. Quercetin 3,7-dimethyl ether 4’-glucoside isolated from the aquatic plant *Nymphoides indica* [8], puerarin V, an isoflavone isolated from the Chinese herb Kudzu (*Pueraria* spp.) root [9] and pilloin, from the stem barks of *Aquilaria sinensis* [7] showed anti-inflammatory activity in diverse models. For example, the effect of quercetin 3,7-dimethyl ether 4’-glucoside on diverse inflammatory markers after ultraviolet B irradiation of keratinocytes (HaCaT) was evaluated [8]. The authors reported that this flavonoid could inhibited p38, JNK, ERK (extracellular-signal-regulated kinase) and IκBα phosphorylation and inhibits the NF-κB expression. In addition, the compound enhanced the activation of skin barrier factors such as filaggrin, involucrin, loricrin and hyaluronic acid synthase-1 (HAS-1) upregulating the expression of these proteins and enzyme. According to these results, the authors [8] suggested that quercetin 3,7-dimethyl ether 4’-glucoside is essential for retaining water and maintaining intercellular space playing an important role in skin moisture retention.

The cardioprotective effect of puerarin V was evaluated in vivo on the isoproterenol (ISO)-induced myocardial infarction (MI) mice and in vitro evaluated in human coronary artery endothelial cells (HCAECs) after induction by LPS [9]. In vivo, puerarin-V improved ventricular wall infarction, decreased the incidence of mortality, inhibited the levels of myocardial injury markers and blocked the upregulation of proinflammatory cytokines (TNF-α, IL-1β and IL-6). In vitro, puerarin-V was able to suppress the activation of *p*-NF-κB. *p*-IκBα and *p*-IKKα/β expression in LPS-induced HCAECs, in a dose dependent manner. Moreover, the same compound increased Bcl-2 (B-cell lymphome 2) expression in LPS-induced HCAECs, whereby puerarin V protected these cells against LPS-induced apoptosis. Overall, the authors [9] considered that this compound can be a new therapeutic option for the treatment of myocardial infarction. Pilloin, isolated from *Aquilaria sinensis* and in bacterial LPS-induced RAW 264.7 macrophages inhibited NF-κB activation, JNK, ERK and p38, reduced the phosphorylation of IκB and suppressed the production of TNF-α, IL-6, COX-2 and iNOS [7].

The chalcone phloretin was studied by Cheon et al. in order to investigate the anti-acne activity against *Propionibacterium acnes*-induced skin infection and the potential target proteins involved in the anti-inflammatory and antibacterial effects. Phloretin decreased the levels of JNK and was able to bind to *P. acnes* β-ketoacyl acyl carrier protein (ACP) synthase III (KAS III), enzyme important in fatty acid synthesis, reducing KAS III-mediated 3-ketoacyl ACP production. This mechanism lead the authors [10] to consider the importance of that chalcone in the prevention and treatment of *P. acnes* infection.

Laavola et al. studied the effect of pine stilbenoids (monomethyl pinosylvin and pinosylvin) on IL-6 in osteoarthritis. In this study, the authors [11] used human osteoarthritis chondrocytes where the expression of the cartilage matrix components aggrecan and collagen II and IL-6 were studied. The results showed that those stilbenoids increased aggrecan expression and suppressed the IL-6 production. In addition, the authors [11] showed that monomethyl pinosylvin and pinosylvin inhibited NF-κB mediated transcription similar to that of ammonium pyrrolidine dithiocarbamate (PDTC) a NF-κB inhibitor.

Islam et al. found that decursinol angelate, a pyranocoumarin isolated from *Angelica gigas*, was able to inhibit the production of IL-1, IL-4, IL-6, IL-1, TNF-α and MAPK activation in phorbol 12-myristate 13-acetate (PMA)- activated human promyelocytic leukemia (HL-60) and LPS-stimulated (RAW 264.7) macrophages. Moreover, decursinol angelate also inhibited the expression of IL-1β and IL-6, NOX (NADPH oxidase) and iNOS in LPS-stimulated (RAW 264.7) macrophages [12].

Beyond terpenes and polyphenols, the bisbenzylisoquinoline alkaloid fangchinoline, extracted from *Stephaniae tetrandrine* S. Moore also presented anti-inflammatory activity [13]. Fangchinoline reduced the growth of human chronic myeloid leukemia (KBM5) and multiple myeloma cells (U266) and repressed NF-κB and AP-1 (activator protein-1) activation by reduction of phosphorylation of IKK and p65. This study suggests that the alkaloid can act as anti-cancer drug although needing validation in appropriate tumor models [13].

Extracts of plants constituted by several components were also evaluated. For example, a crude extract of *Scoparia dulcis* generally used to relieve discomfort caused by painful conditions was studied [14] in terms of anti-inflammatory and analgesic activities in an experimental model of osteoarthritis. This pathology was induced in Wistar rats. This study revealed that the crude extract by intra-articular administration of sodium mono-iodoacetate. After fifteen days of treatment, the crude extract reduced the pro-inflammatory cytokines (IFN-γ [interferon-γ] and IL-6) in the synovial fluid as well as the edema, spontaneous pain and peripheral nociceptive activity [14]. Inhibition of COX-2 at 50 μg/mL was also observed. The chemical composition of the extract was revealed to be particularly rich in polyphenols. The molecular docking analysis showed that all of the polyphenols identified in the extract had high parameter affinity with COX-2 structure, with great emphasis for suspensaside and nicotiflorin, similar to that of meloxicam, a commercial non-steroidal anti-inflammatory drug [14].

An ethyl acetate extract of *Moringa oleifera* was able to reduce the production of pro-inflammatory mediators by LPS-activated human monocyte-derived macrophages (MDM), suppressing mRNA expression of IL-1, IL-6, TNF-α, PTGS2 (prostaglandin-endoperoxide synthase 2, COX-2), NF-κB (p50) and RelA, inhibiting the expression of IL-6, TNF-α and COX-2. These effects lead to the inhibition of phosphorylation of IκB-α and the ability to reduce expression of NF-κB p65, suppressing its nuclear translocation. The composition of this extract with anti-inflammatory activity was 3,4-methyleneazelaic acid; (2*S*)-2-phenylmethoxybutane-1,4-diol; (2*R*)-2-phenylmethoxybutane-1-4-diol; γ-diosphenol; 2,2,4,4,-tetramethyl-6-(1-oxobutyl)-1,3,5-cyclohexanetrione; 3-hydroxy-β-ionone and tuberonic acid [15].

*Eurycoma longifolia* Jack is a wild shrub from Southeast Asian countries described as possessing anti-malarial, anti-cancer and anti-inflammatory activities [15]. The authors demonstrated that the roots of this plant species, constituted by piscidinol A, 24-*epi*-piscidinol A, bourjotinolone and scopoletin, had capacity for suppressing the levels of NO and inhibiting the expression of IL-6, NF-κB and iNOS in LPS-stimulated RAW264.7 cells in a dose-dependent manner [16].

*Tradescantia albiflora* Kunth has been used in Taiwan in traditional medicine for treating hyperuricemia and gout. From the whole plant were isolated diverse compounds some of them being reported for the first time in this plant species—rosmarinosin B, 5-*O*-acetyl bracteanolide, both butenolides and 2β-hydroxyisololiolide, an apocarotenoid. The authors [17] also synthesized four butenolides as novel derivatives of bracteanolide A. In LPS-stimulated RAW 264.7 cells, the butenolide derivative *n*-butyl bracteanolide A showed higher NO inhibitory production than bracteanolide A isolated from the plant [17].

Essential oils have been described as possessing in vitro and in vivo anti-inflammatory activities [18]. The authors studied the effect of the essential oil of oregano—mainly constituted by carvacrol—on the NADPH oxidase mediated oxidative stress and the anti-inflammatory activity using LPS-induced RAW264.7 macrophages. In this cellular system, the oregano essential oil was able to inhibit the expression and secretion of IL-1β, IL-6 and TNF-α and to reduce the activation of MAPK, IκB and NF-κB. Moreover, oregano essential oil also inhibited the LPS-induced RAW264.7 cell inflammation through the NADPH oxidase-mediated production of reactive oxygen species (ROS) [18].

Secretory A2 phospholipases (sPLA2) is a pro-inflammatory protein involved in the mobilization of arachidonic acid by an indirect activation of cytosolic phospholipase A2 (cPLA2) and phospholipase C (PLC). sPLA2 from snake (*Crotalus durissus terrificus*) venom has structural similarity to that of human origin whereby Santos Júnior [19] used a bioaffinity-guided ultrafiltration method on *Crotalus durissus terrificus* sPLA2 in order to detect new anti-inflammatory compounds of diverse origin. In this case, the authors [19] used compounds isolated from *Moquiniastrum floribundum* (Asteraceae) leaf for evaluating their ability for neutralizing the inflammatory activity of sPLA2 from *Crotalus durissus terrificus*. From diverse extracts using organic solvents, the methanol one was that better inhibit sPLA2. Such extract had various compounds but the approach (bioaffinity technique) followed by the authors permitted them to conclude that the flavone hispidulin was able to inhibit the edematogenic and myotoxic activity of sPLA2 but not a decrease in sPLA2 [19].

Not only plants may possess anti-inflammatory properties, wild mushrooms, such as *Echinodontium tinctorium*, also was reported as possessing this activity [20]. A water soluble polysaccharide (AIPetinc) obtained from this mushroom with an average molecular weight of 5kDa and mainly constituted by glucose and with small percentages of galactose, mannose, fucose and xylose was able to restore the histamine-induced inflammatory process in mouse gluteus maximus muscle [20].

In traditional medicine, herbal formulations are frequently used for treating some diseases. Tsang et al. used a formulation with five traditional Chinese herbal medicines (Lonicerae Flos, Menthae Herba, Phellodendri Cortex, Moutan Cortex and Atractylodis Rhizoma) to study its influence on gut microbiota in allergic asthma. The authors [21] proposed this approach because there a correlation between allergic asthma and gut microbiota has been seen upon a change of diet. The formulation after intragastrical administration altered the gut microbial diversity and the short chain fatty acids content in the gut in asthmatic mice. The results also showed that this formulation suppressed pulmonary eosinophilia and asthma-related IL-4 and IL-33 in bronchoalveolar lavage fluid of ovalbumin-induced allergic asthmatic mice. According to the authors [21], the formulation ameliorates symptoms of allergic diseases and may prevent the development of further atopic disorder (allergic asthma) by influencing the gut microbiota.

Three plants (*Aucklandia lappa* DC., *Terminalia chebula* Retz and *Zingiber officinale* Roscoe) have been used in traditional medicine in east Asia to treat chronic diarrhea and abdominal pain [22]. These authors studied the anti-inflammatory activity of these three plants-based formulations in a mouse model of dextran sodium sulfate-induced ulcerative colitis. The anti-inflammatory mechanism was assessed using LPS-treated RAW264.7 macrophages. In this in vitro system, the formulation was able to inhibit NO, IL-6, monocyte chemotactic protein 1 (MCP-1) and TNF-α. The authors [22] attributed the inhibitory effect of the formulation to the reduction of Akt phosphorylation in the in vitro LPS-treated cells. In vivo system, the orally administered formulation also suppressed the expression of TNF-α, IL-6 and MPO in the dextran sodium sulfate-induced ulcerative colitis, beyond symptom improvement (disease activity index, colon length and colon weight).

A review article by Lepanto et al. [23] compiled information on the role of lactoferrin, a cationic glycoprotein able to chelate two ferric irons per molecule and synthesized by exocrine glands and neutrophils, against aseptic (anemia of inflammation, preterm delivery, Alzheimer’s disease and diabetes type 2) and septic inflammation (Chlamydia trachomatis infection, cystic fibrosis and inflammatory bowel disease). The authors [23] reported that orally administered lactoferrin decreases the serum IL-6, which means a reversion of iron homeostasis disorders. Nevertheless, after the revision made by the authors, they also found contradictory in vitro results, ranging from pro- to anti-inflammatory activity of lactoferrin depending on the cell models used, cell metabolic status, stimulatory or infecting agents and finally to the iron saturation degree of lactoferrin, its integrity and purity [23].

It is expected that this Special Issue will promote interest in the search for natural products as potential anti-inflammatory agents.
